# PI3K-Dependent GSK3ß(Ser9)-Phosphorylation Is Implicated in the Intestinal Epithelial Cell Wound-Healing Response

**DOI:** 10.1371/journal.pone.0026340

**Published:** 2011-10-19

**Authors:** Thomas Karrasch, Tanja Spaeth, Brigitte Allard, Christian Jobin

**Affiliations:** 1 Department of Internal Medicine I, University of Regensburg, Regensburg, Germany; 2 Department of Medicine and Center for Gastrointestinal Biology and Disease, University of North Carolina at Chapel Hill, Chapel Hill, North Carolina, United States of America; 3 Department of Pharmacology, University of North Carolina at Chapel Hill, Chapel Hill, North Carolina, United States of America; Cardiff University, United Kingdom

## Abstract

**Introduction:**

The ability of the intestinal epithelial barrier to respond to various injurious insults is an essential component of intestinal homeostasis. However, the molecular mechanisms responsible for wound-healing and repair in the intestine are poorly understood. The glycogen synthase kinase 3ß (GSK3ß) has been implicated in various biological processes such as cellular motility, cell spreading and recently inflammation.

**Aim:**

To investigate the role of GSK3ß in intestinal epithelial cell restitution.

**Methods:**

Rat intestinal epithelial IEC18 cells were serum-starved for 16 to 24h and wounded by multiple scraping. Akt(Ser473)-, GSK3ß(Ser9)- and RelA(Ser536)-phosphorylation were determined by Western blot using specific phospho-antibodies. The inhibitors AG1478 (1 µM) and Ly294002 (25 µM) were used to block EGF-R autophosphorylation and PI3K-activation, respectively. ß-catenin/LEF/TCF dependent transcription was determined by reporter gene assay (TOP/FOP system). C-myc gene expression was evaluated by real-time RT-PCR. GSK3ß^−/−^ mouse embryonic fibroblasts were used to characterize the role of GSK3ß in wounding-induced cell migration.

**Results:**

Wounding induced GSK3ß(Ser9) phosphorylation in IEC-18 cells, which led to ß-catenin accumulation as well as nuclear translocation of ß-catenin. ß-catenin stabilization/nuclear translocation led to enhanced LEF-TCF transcriptional activity and subsequent c-myc mRNA accumulation in wounded cell monolayers. Blocking PI3K/Akt signaling with Ly294002 prevented wound-induced GSK3ß(Ser9) phosphorylation as well as ß-catenin nuclear translocation and significantly attenuated restitution. Additionally, wounding induced rapid NF-kB(Ser536) phosphorylation, which was inhibited by AG1478, but not by Ly294002. GSK3ß^−/−^ cells demonstrated significantly attenuated wound-induced restitution compared to wild-type cells.

**Conclusion:**

We conclude that PI3K-mediated GSK3ß phosphorylation is involved in the intestinal epithelial wound-healing response. Phosphorylation of GSK3ß may be important for intestinal restitution by promoting cell motility in response to wounding.

## Introduction

The gastrointestinal tract of higher organisms is lined by a monolayer of intestinal epithelial cells providing a barrier against the unregulated translocation of various luminal antigens to the mucosal lamina propria, which could lead to an undesirable immune cell activation and inflammation. Acute breaches within the epithelial monolayer necessitate a rapid and efficient resealing of the resulting gap mediated by adjacent cells [1], [2], [3].

Both host-derived factors from the interstitium (various growth factors and cytokines) as well as factors generated within the intestinal environment (bile acids, short chain fatty acids and luminal microbial products) support this restitution response [4], [5]. At the molecular level, these mediators induce multiple signaling events within intestinal epithelial cells, including NF-kB-, MAPKp38-, TAK1-, FAK-activation via Smad2/3 and Akt-activation via PI3K and ErbB4. In turn, these signaling events modulate wound-healing responses through anti-apoptotic, pro-proliferative as well as pro-migratory effects [6], [7], [8], . Remarkably, many of these signaling mechanisms can also be induced independent of external stimuli by mechanical wounding of the intestinal epithelial cell monolayer [11], [12].

Recently, the glycogen synthase kinase 3ß (GSK3ß) pathway has been demonstrated to modulate cell spreading and migration upstream of focal adhesion kinase (FAK) in fibroblasts in vitro [13]. In addition, GSK3ß phosphorylation downstream of the small GTPase Cdc42 modulates cell migration in astrocytes [14]. GSK3 activity has been implicated in the modification of the apical junctional complex between adjacent enterocytes during intestinal epithelial epidermal-mesenchymal transition (EMT) [15], [16]. Importantly, hydrogen peroxide supports intestinal epithelial Caco2 cell migration via PI3K-dependent FAK activation [17], and interferon-gamma regulates intestinal epithelial cell homeostasis via the ß-catenin signaling pathway [18]. These results place the GSK3ß/ß-catenin/T-cell factor/lymphoid enhancer factor (TCF/LEF) signaling cascade at the forefront of gastrointestinal epithelial homeostasis in health and disease.

However, GSK3ß's role during mechanical wounding-induced enterocyte wound-healing has not been well defined. We used rat intestinal epithelial IEC18 cells grown to confluency as a well-established in vitro model of the gastrointestinal wound-healing response [12], [19], [20], [21], [22], [23]. Here, we show that wound-induced IEC18 cell restitution triggered PI3K-dependent GSK3ß-phosphorylation at position Ser9, followed by accumulation and nuclear translocation of ß-catenin, TCF/LEF-dependent gene expression and the accumulation of c-myc mRNA in these cells. Blocking PI3K-activation significantly attenuated GSK3ß phosphorylation as well as wounding-induced IEC18 cell monolayer restitution.

## Materials and Methods

### Cell culture wounding, migration and treatment

The non-transformed rat ileal epithelial cell line IEC18 (American Type Culture Collection (ATCC) CRL1589, Manassas, VA) was used between passages 8 and 20. Cells were grown to confluency in 6-well plates (Costar, Corning Inc, Acton, MA), starved overnight in serum-reduced media (1% FCS), and then standardized wounding was performed by multiple linear scraping with a P1000 pipet tip as described previously [12], [19], [20], [21], [22], [23]. The pharmacological inhibitors AG1478 (from Calbiochem, San Diego, CA, USA) and Ly294002 (from Merck Biochemicals, Darmstadt, Germany) were dissolved in dimethyl sulfoxide (DMSO; Sigma, St Louis, MO) to a final concentration of 1 µM and 25 µM, respectively. 25 µM Ly294002 effectively inhibited PI3K signaling in preliminary dose-titration experiments in IEC18 cells, and a dose of 20–25 µM has been widely used in previous studies in IEC6 and IEC18 cell lines [24], [25], [26]. Where indicated, cells were pretreated with these inhibitors or solvent control for 30 minutes before being wounded by multiple scraping for the times indicated.

For cell migration experiments, cells were grown in 100 mm cell culture dishes (Falcon, BD Biosciences, Franklin Lakes, NJ), and standardized wounds were created using a razor blade. Migration over the wound-edge was determined over time as described previously [11], [12], [20], [27]. GSK3ß^−/−^ and GSK3ß^wt/wt^ mouse embryonic fibroblasts used in cell migration experiments were a generous gift of Jim Woodgett (Samuel Lunenfeld Research Institute, Mount Sinai Hospital, Toronto, Canada) and have been described previously [28].

### IEC18 cell plasmid transfection and TOP/FOP-luciferase reporter assay

IEC18 cells were cultured to confluency, after which they were transfected with a plasmid carrying a luciferase reporter system under the TOP/FOP promoter using FuGene HD transfection reagent (from Roche Diagnostics, Mannheim, Germany) in serum-free media (Opti-MEM; Gibco, Grand Island, NY) according to the manufacturer's instructions. After 12 h, cells were washed, fresh serum-reduced media (1%) was added and cells were subjected to standard multiple scrape-wounding. The plasmid containing a TCF/LEF-dependently expressed luciferase reporter (a generous gift of Bert Vogelstein, John Hopkins University School of Medicine, Baltimore, MD) has been described previously [29].

Cells were lysed using cell culture lysis reagent (from Promega, Madison, WI, USA) 13 hours and 24 hours after wounding, respectively, and luciferase activity was determined in cell lysates using a Luciferase Assay System (from Promega, Madison, WI, USA) as described by the manufacturer. Light emission readings were performed using the Varioskan Flash Reader (from Thermo Scientific, Waltham, MA) according to the manufacturer's instructions. Values were normalized to total protein content in cell lysates generated from similarly treated wells (mean values of 3 wells) as determined using the BCA Protein Assay (from Thermo Scientific, Waltham, MA).

### RNA extraction and amplification by RT-PCR

RNA was isolated using TRIzol method (Invitrogen, Carlsbad, CA), reverse transcribed (1 µg RNA) and amplified as previously described [12], [30] using primers specific for c-myc and GAPDH. For semi-quantitative assessment of mRNA accumulation, real-time PCR was performed using SYBR Green fluorescent dye in the LightCycler system (from Roche Applied Science, Mannheim, Germany). Primer sequences were as follows: rat c-myc, forward 5′-AGTGCATCGACCCCTCAGT-3′ and reverse 5′-TCAATTTCTTCCTCATCATCTTGT-3′; rat GAPDH, 5′-CCCTCTGGAAAGCTGTGG-3′ and 5′-GCTTCACCACCTTCTTGATGT-3′.

### Western blot analysis and subcellular fractionation

Wounded, stimulated or unstimulated cells were collected, lysed in RIPA buffer, and the protein concentration was measured using BCA protein quantification assay (from Thermo Scientific, Waltham, MA). Protein extracts (20 µg) were further dissolved in 1× Laemmli buffer, subjected to electrophoresis on a 10% SDS-PAGE and transferred to nitrocellulose membranes. Antibodies recognizing phospho-Akt(Ser473), phospho-GSK3ß(Ser9), phospho-RelA(Ser536), ß-catenin and GAPDH were from Cell Signaling (Cell Signaling Technology Inc, Beverly, MA). All antibodies were used at a 1:1000 dilution in a solution containing 5% BSA in TBS-T. Immunoreactive proteins were detected using the enhanced chemiluminescence light (ECL) detecting kit (Amersham Biosciences, Piscataway, NJ) as described previously [12], [30].

Subcellular fractionations were performed using specific cell lysis buffers and repeated centrifugation steps (Nuclear extract kit from Active Motive, Carlsbad, CA) according to the manufacturer's specifications. Cell fractions were dissolved in RIPA buffer, and the protein concentration was measured using BCA protein quantification assay. Protein extracts (20 µg) were further dissolved in 1× Laemmli buffer, subjected to electrophoresis on a 10% SDS-PAGE and transferred to nitrocellulose membranes as described above. Western blot analysis of poly-ADP-ribose polymerase (PARP, from Cell Signaling Technology Inc, Beverly, MA) and superoxide dismutase 2 (SOD2, from ABFrontier, Seoul, Korea) were performed to confirm appropriate separation of nuclear and cytosolic proteins.

### Statistical analysis

Unless otherwise indicated, data are representative of three independent experiments. In vitro restitution data are expressed as median and interquartile range (IQR) of nine separate wounds per condition. Luciferase measurements are expressed as median and interquartile range (IQR) of 3–4 independent experiments, each with three separate wounds/dishes per condition and were adjusted for total protein content of the respective cell lysates as described above. C-myc mRNA accumulation relative to control is expressed as median and interquartile range (IQR) of three separate experiments, each with three separate wells per condition. Statistical analysis was performed by the Kruskal-Wallis test for non-parametric data using the SPSS software (SPSS Inc., Chicago, IL, USA), and differences were considered significant if p values were <0.05. Densitometry was performed for all Western blots and was normalized to the respective loading controls (GAPDH, SOD, PARP). Protein expression is expressed as -fold protein induction relative to the respective unwounded control. Data are expressed as mean ± S.D. of 3–4 independent experiments per condition. Statistical analysis was performed by the two-tailed Student's *t* test using the SPSS software (SPSS Inc., Chicago, IL, USA), and differences were considered significant if p values were <0.05.

## Results

### Wounding induces GSK3ß(Ser9)-phosphorylation and leads to ß-catenin accumulation and nuclear translocation in intestinal epithelial cell monolayers

**Figure 1:**
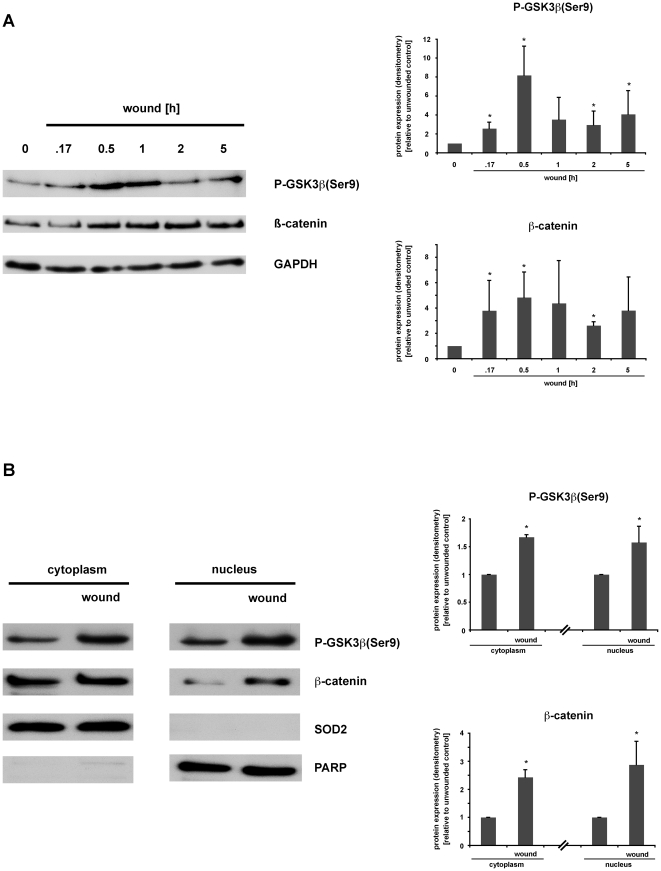
Wounding induces GSK3ß(Ser9) phosphorylation, ß-catenin accumulation and nuclear translocation in IEC18 cell monolayers. Rat intestinal epithelial IEC18 cells were grown to confluency in 6-well-plates, serum-starved overnight, and cell monolayers were then wounded by multiple scraping. (A) At the indicated time points, total cell protein extracts were prepared and analysed for GSK3ß signaling pathway activation via Western blot. (B) 30 minutes after wounding, cells were lysed, subcellular fractions of nuclear/cytoplasmic protein were prepared and analysed for phospho-GSK3ß(Ser9) and ß-catenin accumulation via Western blot. (A,B) Densitometry was performed on n = 3–4 different Western blots (each representative of an independent experiment) per condition and normalized to the respective loading controls (GAPDH, SOD, PARP). Protein expression is expressed as -fold protein induction relative to the respective unwounded control. Data are expressed as means ± S.D. of 3–4 different experiments per condition. Statistical analysis was performed by the two-tailed Student's *t* test (*p<0.05 versus control).

We first examined the impact of mechanical wounding on GSK3ß signaling in the rat IEC18 cell monolayers. Mechanical wounding rapidly induced GSK3ß-phosphorylation at position Ser9 (Fig. 1A). In addition, accumulation of ß-catenin protein, the down-stream target of GSK3ß, was enhanced in wounded IEC18 cells (Fig. 1A).

We next determined the subcellular localization of ß-catenin using cytoplasmic and nuclear extracts. As expected, ß-catenin was predominantly localized within the cytosolic fraction in resting cells (Fig. 1B). However, mechanical wounding induced the accumulation of ß-catenin within the nuclear fraction in IEC18 cell monolayers (Fig. 1B). In summary, scrape-wounding induces GSK3ß phosphorylation at position Ser 9, leading to ß-catenin accumulation as well as its nuclear translocation in intestinal epithelial cells.

### Mechanical wounding induces TCF/LEF-dependent gene expression in IEC18 cell monolayers

**Figure 2:**
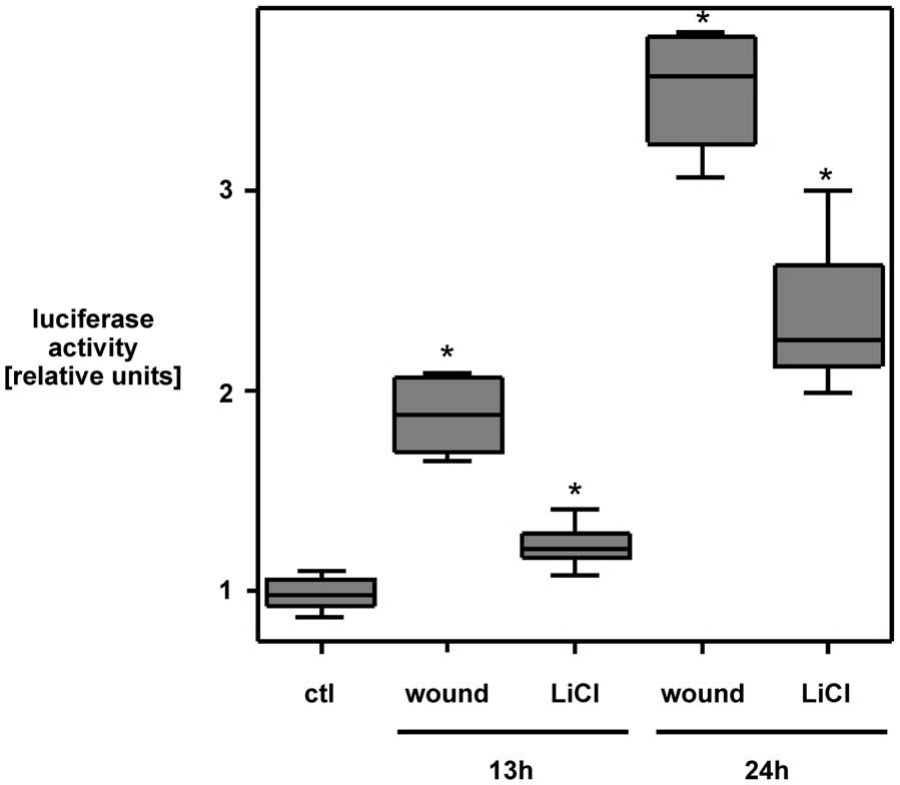
Wounding strongly induces TCF/LEF-dependent gene transcription in IEC18 cell monolayers as compared to control-treated cells. IEC18 cells were grown to confluency, transfected with a TOP/FOP-reporter plasmid (expressing the luciferase gene under the TCF/LEF promoter) and then wounded by multiple scraping. Cell extracts were prepared after 13 h and 24 h, and luciferase activity was determined in cell lysates and normalized for protein content in the respective wells. The GSK3ß inhibitor Lithium Chloride (LiCl, 20 mmol/l) was used as positive control. Data are expressed as median and interquartile range (IQR) of n = 3–4 independent experiments, each with three separate wounds/dishes per condition, and luciferase activity was adjusted for total protein content of the respective cell lysates. Statistical analysis was performed by the Kruskal-Wallis test for non-parametric data (*p<0.05 versus control). Wounding led to a strong, time-dependent induction of luciferase activity as compared to control-treated cells, indicating TCF/LEF-dependent gene transcription.

We next investigated the functional impact of ß-catenin nuclear translocation on transcriptional activation. In accordance with increased nuclear ß-catenin, scrape-wounding time-dependently induced TCF/LEF-dependent luciferase activity in IEC18 cell monolayers compared to control-treated cells (Fig. 2).

**Figure 3:**
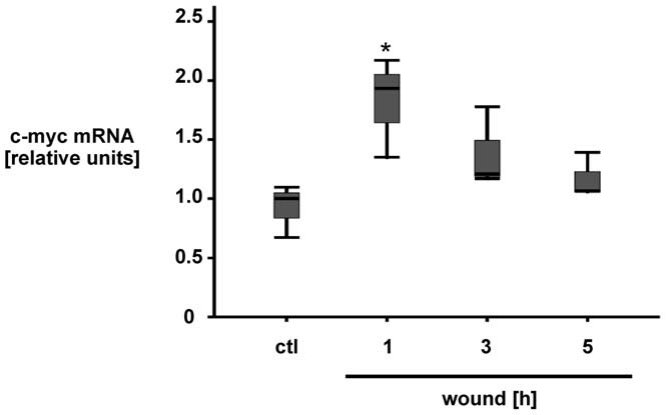
Wounding induces c-myc mRNA accumulation in IEC18 cell monolayers. Rat intestinal epithelial IEC18 cells were grown to confluency in 6-well-plates, serum-starved overnight, and cell monolayers were then wounded by multiple scraping. At the indicated time points, total cell RNA extracts were prepared and analysed for c-myc mRNA accumulation normalized to GAPDH control via real-time RT-PCR using specific primers. mRNA accumulation relative to unwounded control is expressed as median and interquartile range (IQR) of n = 3 separate experiments, each with three separate wells per condition. Statistical analysis was performed by the Kruskal-Wallis test for non-parametric data (*p<0.05 versus control).

C-myc gene expression has been characterized as a down-stream target of ß-catenin signal transduction, increasing intestinal epithelial cell survival and proliferation [31], [32]. Thus, to determine the functional relevance of increased TCF/LEF-dependent luciferase activity we measured c-myc mRNA in wounded IEC18 cell monolayers. Interestingly, scrape-wounding induced significant accumulation of c-myc mRNA in IEC18 cells (Fig. 3). In summary, scrape-wounding induces TCF/LEF-dependent gene transcription as well as c-myc mRNA accumulation in confluent intestinal epithelial cell monolayers.

### PI3K signaling is important for wound-induced GSK3ß-phosphorylation and ß-catenin nuclear translocation

**Figure 4:**
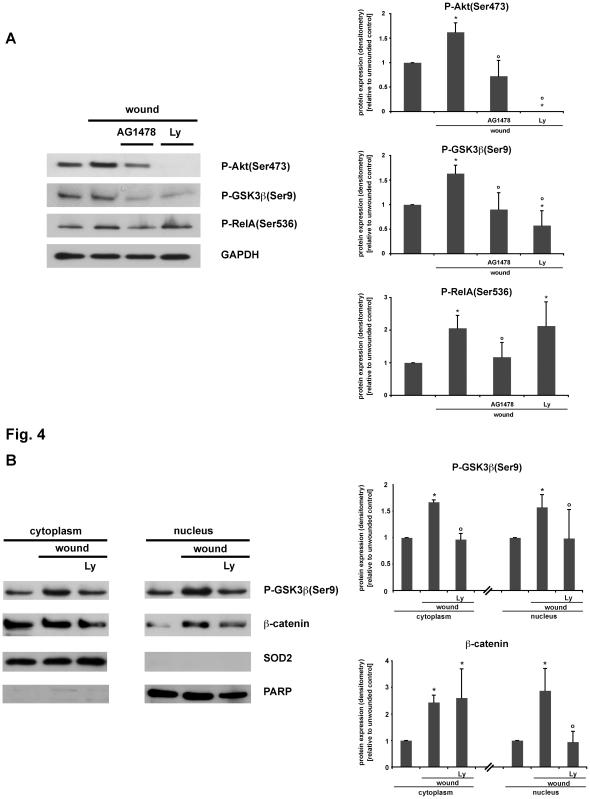
Wounding-induced GSK3ß(Ser9) phosphorylation and ß-catenin nuclear translocation is dependent on PI3K activation. Confluent IEC18 cell monolayers were pretreated for 30 minutes with the specific inhibitors of PI3K activation Ly294002 (25 µM, Ly) and EGF-R autophosphorylation AG1478 (1 µM) or solvent control and then wounded by multiple scraping. (A) Total cell protein extracts were prepared 30 minutes after wounding and analysed via Western blot. Wounding-induced Akt(Ser473)- and GSK3ß(Ser9)-phosphorylation were blocked by Ly294002 as well as reduced by AG1478. As positive control, AG1478 caused inhibition of RelA phosphorylation (indicating NF-kB activation) after wounding is shown [11]. (B) 30 minutes after wounding, cells were lysed, subcellular fractions of nuclear/cytoplasmic protein were prepared and analysed for phospho-GSK3ß(Ser9) and ß-catenin accumulation via Western blot. PARP and SOD2 were only detected in the nuclear or cytoplasmic protein fractions, respectively. (A,B) Densitometry was performed on n = 3–4 different Western blots (each representative of an independent experiment) per condition and normalized to the respective loading controls (GAPDH, SOD, PARP). Protein expression is expressed as -fold protein induction relative to the respective unwounded control. Data are expressed as means ± S.D. of 3–4 different experiments per condition. Statistical analysis was performed by the two-tailed Student's *t* test (*p<0.05 versus unwounded control, **°**p<0.05 versus wounded cells).

PI3K has been described to induce phosphorylation of Akt at position Ser473, thereby mediating the phosphorylation of GSK3ß at position Ser9. This in turn inactivates GSK3ß kinase activity [33], [34]. Of note, we observed a significant induction of Akt phosphorylation in response to scrape-wounding IEC18 cell monolayers (Fig. 4A). Thus, we investigated the role of PI3K in wounding-induced GSK3ß phosphorylation. Interestingly, pretreatment with the pharmacological PI3K inhibitor Ly294002 abolished wounding-induced Akt(Ser473)- as well as GSK3ß(Ser9)-phosphorylation in IEC18 cell monolayers (Fig. 4A).

It has previously been demonstrated that wounding induces EGF-R autophosphorylation in intestinal epithelial cells, leading to RelA(Ser536)-phosphorylation and NF-kB-dependent gene transcription [11]. Of note, AG1478 (a specific inhibitor of EGF-R autophosphorylation) prevented wounding-induced RelA(Ser536) phosphorylation and reduced wounding-induced Akt(Ser473)- as well as GSK3ß(Ser9)-phosphorylation (Fig. 4A). We next determined the impact of PI3K signaling on wounding-induced ß-catenin nuclear translocation. As seen in fig. 4B, Ly294002 abrogated wounding-induced ß-catenin nuclear translocation in IEC18 cell monolayers. These findings suggest that PI3K signaling is involved in wounding-induced GSK3ß(Ser9) phosphorylation and ß-catenin nuclear translocation in IEC18 intestinal epithelial cells.

### PI3K signaling is important for wound-induced cell migration. GSK3ß^−/−^ mouse embryonic fibroblasts exhibit reduced cell migration in response to scrape wounding

**Figure 5:**
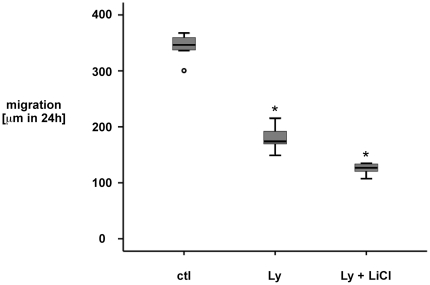
IEC18 cell monolayer restitution in response to wounding is dependent on PI3K signaling. Confluent IEC18 cell monolayers were pretreated for 30 minutes with the specific inhibitor of PI3K activation Ly294002 (Ly, 25 µM) or solvent control, standardized wounds were created using a razor blade and restitution was monitored over 24 h using serial microphotography. In a separate set of experiments, the GSK3ß inhibitor Lithium Chloride (LiCl, 20 mmol/l, pretreatment of an additional 30 minutes before exposure to Ly) was used to inhibit GSK3ß kinase activity in Ly294002-treated cells. Ly294002 significantly inhibited wounding-induced restitution as compared to control-treated cells, which was not reversed by LiCl-treatment. Data are expressed as median and interquartile range (IQR) of n = 9 independent wounds per condition. Statistical analysis was performed by the Kruskal-Wallis test for non-parametric data (*p<0.05 versus control).

GSK3ß phosphorylation has been implicated in cell spreading and cell migration in various cellular systems [13], [14],[35],[36]. We next determined the role of PI3K activation in wounding-induced IEC18 cell restitution. Of note, Ly294002 exposure significantly decreased wounding-induced cell restitution in IEC18 cells (Fig. 5). Remarkably, adding the GSK3ß inhibitor Lithium Chloride (LiCl, 20 mmol/l) to wounded cell monolayers was not able not reverse the effect of Ly294002 treatment (Fig. 5). These findings suggest that PI3K signaling is involved in wounding-induced cellular restitution in IEC18 intestinal epithelial cells. However, the effect of Ly294002 treatment seems to be independent of GSK3ß activity.

**Figure 6:**
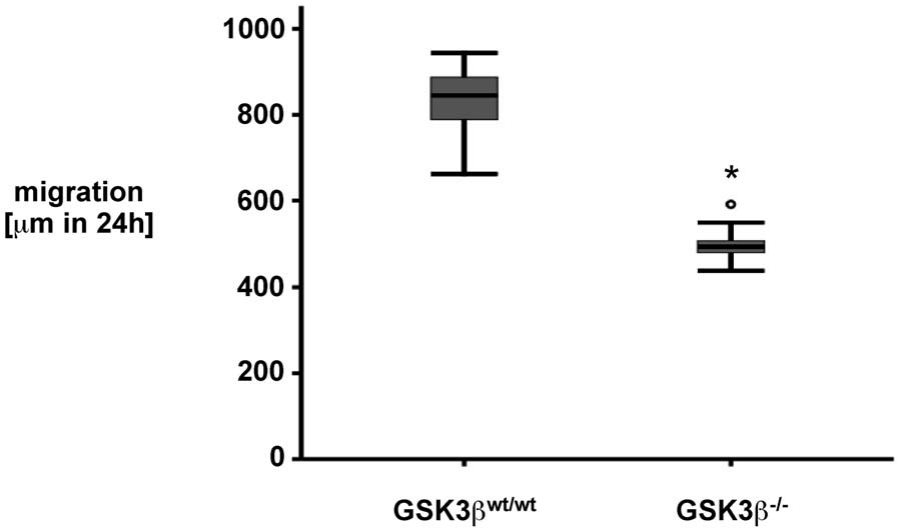
GSK3ß^−/−^ mouse embryonic fibroblasts display reduced restitution in response to scrape-wounding as compared to GSK3ß^wt/wt^ cells. Mouse embryonic fibroblasts (MEF) were prepared from GSK3ß^−/−^ and GSK3ß^wt/wt^ mice and grown to confluency. Cells were serum-starved overnight, standardized wounds were created using a razor blade and restitution was monitored over 24 h using serial microphotography. Data are expressed as median and interquartile range (IQR) of n = 9 independent wounds per condition. Statistical analysis was performed by the Kruskal-Wallis test for non-parametric data (*p<0.05 versus control).

To extend the findings obtained with a pharmacological inhibitor, we performed functional migration assays using GSK3ß^−/−^ mouse embryonic fibroblasts (MEF). These GSK3ß^−/−^ MEF exhibit ß-catenin levels comparable to GSK3ß^wt/wt^ MEF controls [28], [37]. Interestingly, cellular migration of GSK3ß^−/−^ MEF compared to GSK3ß^wt/wt^ MEF was significantly reduced (Fig. 6). Of note, Takada et al demonstrated that GSK3ß^−/−^ MEF were defective in TNF-induced Akt phosphorylation [38]. This finding indicates that complete ablation of GSK3ß impairs cell migration, however, the association of PI3K, Akt, GSK3ß, ß-catenin and cell migration proves to be complex.

## Discussion

Genetic and pharmacological studies have highlighted the key role of a functional intestinal barrier in maintaining gastrointestinal epithelial homeostasis and preventing the development of inflammatory bowel diseases [39], [40], [41]. Since the intestinal epithelial layer is in a continual state of stress through a constant exposure to dietary and microbial toxins, medication and physically noxious agents, a proper wound-healing response is essential to maintain a functional epithelial barrier. So far the molecular events responsible for intestinal epithelial cells' wound healing response are poorly defined. In this study, we identified PI3K and GSK3ß as key signaling molecules involved in the intestinal epithelial wound-healing response.

The lipid PI3K has been demonstrated to be involved in various signaling pathways modulating cell metabolism, cell proliferation and survival. Upon activation, PI3K is known to induce Akt phosphorylation via PDK1 activation using PIP3 as a second messenger [42]. Akt in turn phosphorylates GSK3ß at position Ser 9, leading to its inactivation [33], [34]. Interestingly, we found a rapid phosphorylation of GSK3ß at position Ser 9 in confluent intestinal epithelial IEC18 cell monolayers in response to wounding, followed by an accumulation and nuclear translocation of ß-catenin as well as an induction of TCF/LEF-dependent transcriptional activity (Fig. 1; Fig. 2; Fig. 3). Pretreatment with the specific PI3K inhibitor Ly294002 abolished wounding-induced GSK3ß(Ser9) phosphorylation in IEC18 cell monolayers and inhibited ß-catenin nuclear translocation, which is a downstream effect of GSK3ß phosphorylation, in wounded IEC18 cell monolayers (Fig. 4). These results indicate a PI3K-dependent activation of the GSK3ß/ß-catenin/T-cell factor/lymphoid enhancer factor (TCF/LEF) signaling cascade in wounded intestinal epithelial cell monolayers.

Ly294002 significantly attenuated wounding-induced IEC18 cell monolayer restitution, indicating that PI3K activation is necessary for these wound-healing responses (Fig. 5). However, treatment of Ly204002-exposed wounded cell monolayers with the GSK3ß inhibitor Lithium Chloride (LiCl, 20 mmol/l) was not able not reverse this effect (Fig. 5). Of note, the class I PI3K are heterodimeric molecules composed of a regulatory and a catalytic subunit named p85 and p110, respectively. They have been demonstrated to impact on cell polarity and cell motility, on cell cycle and cell survival as well as on cell metabolism separately via a variety of independent signaling cascades (Rac/Cdc42, PDK1/Akt/FOXO, PDK1/Akt/GSK3ß) [33]. Thus, the effects observed in response to inhibition of PI3K activity using Ly294002 may only in part depend on PI3K-dependent GSK3ß signaling during enterocyte wound-healing. Of note, it has recently been demonstrated that PI3K-inhibition supports to the formation of the apical junctional complex via GSK3ß activity in a colorectal cancer cell line, thereby inhibiting cell migration in these cells [43]. The precise mechanism responsible for attenuated restitution in Ly294002-treated IEC18 cell monolayers will be the focus of future studies.

Remarkably, cellular migration in scratch-wound assays was significantly reduced in GSK3ß^−/−^ mouse embryonic fibroblasts (MEF) compared to GSK3ß^wt/wt^ MEF (Fig. 6). Of note, GSK3ß^−/−^ MEF and GSK3ß^wt/wt^ MEF have similar ß-catenin levels [28], [37]. Interestingly, GSK3ß^−/−^ MEF are defective in TNF-induced Akt phosphorylation [38]. This finding indicates that complete ablation of GSK3ß impairs cell migration, however, the association of PI3K, Akt, GSK3ß, ß-catenin and cell migration proves to be complex. Other studies have demonstrated the impact of GSK3ß on cell migration to be cell-type- and stimuli-specific [36]: GSK3ß(Ser9) phosphorylation can have both an inhibitory [24], [44] as well as a supporting effect [13], [14], [45] on cell migration. The precise mechanism responsible for the reduced migration of GSK3ß^−/−^ MEF will be the focus of future studies.

**Figure 7:**
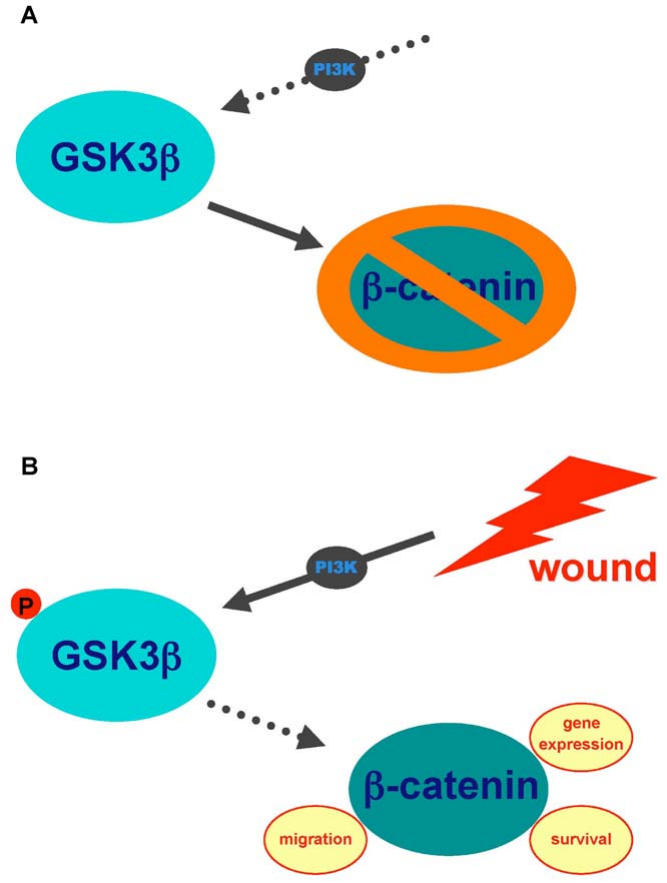
PI3K-dependent GSK3ß(Ser9) phosphorylation is implicated in the intestinal epithelial cell wound-healing response (Schematic representation). (A) In resting intestinal epithelial cell monolayers, PI3K is inactive, and GSK3ß in its unphosphorylated (active) state phosphorylates ß-catenin, leading to its inactivation. (B) Wounding activates PI3K, leading to GSK3ß phosphorylation at position Ser9. This phosphorylation inactivates GSK3ß, leading to ß-catenin accumulation in intestinal epithelial cells. ß-catenin translocates to the nucleus, modulating gene expression, cell survival and potentially cell migration as well.

Our data further characterize GSK3ß's role during immediate intestinal epithelial cell restitution: Wounding IEC18 cell monolayers leads to rapid ß-catenin accumulation and nuclear translocation via PI3K-dependent GSK3ß inhibition through its phosphorylation at position Ser 9. This is followed by TCF/LEF-dependent gene expression and accumulation of c-myc mRNA in wounded cells, which has been demonstrated to increase intestinal epithelial cell survival and proliferation [31], [32] (Fig. 7A, B).

Of note, GSK3ß has been proposed as a molecular target for inflammatory disorders [46], [47], [48], [49]. However, our study suggests that blocking this signaling pathway could have deleterious consequences for the host by impairing epithelial restitution and compromising intestinal barrier function. Further studies measuring intestinal injury responses in mice with epithelial-tissue defective GSK3ß will be necessary to clarify the role of this signaling protein in vivo.
